# Trajectories of Dietary Patterns, Sleep Duration, and Body Mass Index in China: A Population-Based Longitudinal Study from China Nutrition and Health Survey, 1991–2009

**DOI:** 10.3390/nu12082245

**Published:** 2020-07-27

**Authors:** Yingting Cao, Xiaoyue Xu, Zumin Shi

**Affiliations:** 1 Preclinical Diseases and Prevention, Baker Heart and Diabetes Institute, Melbourne, VIC 3004, Australia; tina.cao@unimelb.edu.au; 2 Melbourne School of Population and Global Health, The University of Melbourne, Melbourne, VIC 3000, Australia; 3 School of Public Health and Community Medicine, University of New South Wales, Sydney 2052, Australia; luna.xu@unsw.edu.au; 4 Improving Palliative, Aged and Chronic Care through Clinical Research and Translation, Faculty of Health, University of Technology, Sydney 2007, Australia; 5 Human Nutrition Department, College of Health Sciences, QU Health, Qatar University, Doha 2713, Qatar

**Keywords:** trajectory of dietary patterns, BMI, sleep duration, Chinese adults

## Abstract

No study has used trajectories of dietary patterns to examine their effects on sleep duration and body mass index over time in the Chinese population. We analyzed data from adults participating in the China Health and Nutrition Survey between 1991 and 2009. Dietary intake was measured by a 24-h recall method over three consecutive days. Height and body weight were measured, and sleep duration was self-reported. Multivariable mixed linear models were applied to examine the association between trajectories of dietary patterns (using a latent class model) and sleep duration as well as BMI. Four trajectories of a traditional pattern (characterized by rice, meat, and vegetables) and three trajectories of a modern pattern (characterized by fast food, milk, and deep-fried food) were identified. Participants with a high and rapid increase trajectory of the modern dietary pattern had the shortest sleep duration (β = −0.26; 95% CI: −0.40, −0.13). Participants with a high and stable intake of the traditional dietary pattern had the lowest BMI (β = −1.14; 95% CI: −1.41, −0.87), while the participants with a high and rapid increase trajectory of the modern dietary pattern had the highest BMI (β = 0.74; 95% CI: 0.34, 1,15). A rapid increase in the modern dietary pattern is associated with shorter sleep duration and higher BMI.

## 1. Introduction

Short sleep duration and poor sleep quality are associated with increased cardiometabolic risks such as obesity and diabetes [1]. Short sleep duration is prevalent worldwide [2,3]. In China, approximately a quarter of community-dwelling adults aged 60 years and above reported sleep durations of ≤6 h per day [4,5]. These numbers are comparable to developed countries, but the mechanism of short sleep and association with related chronic conditions such as obesity have not been thoroughly studied in the Chinese population.

There is a growing interest in the relationship between sleep and food intake. As suggested by previous studies, compared to normal sleepers, short sleepers are more likely to have higher energy intakes, in particular from fat and snacks [6,7]. The evidence of the associations between diet and sleep has been summarized in a systematic review [8]. Given the fact that food is consumed in combination and neglecting the interaction between nutrients/foods may generate inconsistent results, dietary patterns have been examined for their association with sleep outcomes in recent years. In conventional dietary pattern analysis, two different approaches are commonly used to derive specific dietary patterns: the hypothesis-oriented approach (such as diet quality scores) and the exploratory method (such as factor analysis or cluster analysis) [9]. By using either hypothesis-oriented or exploratory approaches, studies have reported that a healthy dietary pattern is associated with a short sleep onset [10] and reduced odds of short sleep duration [11]. However, the causality between diet and sleep has yet to be confirmed.

China has experienced a remarkable but undesirable nutrition transition towards a stage of nutrition characterized by a high intake of fat and animal-sourced foods, which has resulted in a high prevalence of diet-related disorders such as obesity, diabetes, and cardiovascular diseases [12]. Longitudinal analysis of dietary transitions in the Chinese population has suggested that the traditional dietary pattern (characterized by rice, pork, and vegetables) has remained stable between 1991 and 2009, while the modern dietary pattern (characterized by wheat products and fast foods) has become more popular over time [13]. In addition, the more dynamic modern dietary pattern has been positively associated with cardiometabolic risks over time in the Chinese population [14]. Fewer studies have examined the associations between dietary patterns and sleep outcomes, as well as BMI, in China. Using data from the China Kadoorie Biobank study, Yu et al. reported that both traditional northern (high in wheat and other staple foods) and modern dietary patterns (high in meat, poultry, fish, and dairy products) were associated with a decreased prevalence of insomnia symptoms [15]. However, they did not investigate the associations between dietary patterns and sleep duration or BMI. More importantly, while most of the studies applied a cross-sectional design, they failed to track trajectories of dietary patterns over time and were not able to examine changes within each dietary pattern and sleep indicators or BMI. Therefore, there is a need to examine the effect of changes in dietary patterns on sleep duration and BMI by using a longitudinal design in order to provide an insight into the associations between diet, sleep, and obesity.

A novel approach of using trajectories of dietary patterns that are based on groups/clusters over time to model longitudinal dietary intake has become popular in the field of nutritional epidemiology. The construction of the trajectory of dietary patterns usually uses “proc traj” in SAS [16]. The method has been used in different populations, including children [17] and adults [18]. However, to our best knowledge, no study has used this approach to construct trajectories of dietary patterns and examine their effects on short sleep and BMI over time in the Chinese population. Using the data from a longitudinal survey, the China Nutrition and Health Survey (CHNS) from 1991 to 2009, the aims of this study are to (1) identify trajectories of dietary patterns over time and (2) examine the association between the trajectories of dietary patterns and sleep duration as well as BMI.

## 2. Materials and Methods

### 2.1. Study Population

CHNS is a population-based longitudinal survey conducted in China, with ten waves so far (1989–2015). It has been widely used to examine the effects of diet on health outcomes over time [19]. The survey used a multistage, random cluster process to select a sample of about 7200 households, with a total of approximately 30,000 individuals in 15 provinces that vary substantially in geography, economic development, public resources, and health indicators. Details of the study design are described elsewhere [14,20]. For the present study, the participants (aged 20 and above) who had attended at least three waves of the dietary survey between 1991 and 2009, with a total of 10,495 adults, were included in the analysis.

The institutional review committees of the University of North Carolina at Chapel Hill and the National Institute of Nutritional and Food Safety, China Centre for Disease Control and Prevention, have approved survey protocols, instruments, and the process of obtaining informed consent for the study [19].

### 2.2. Outcome Measures: Sleep Duration and BMI

Sleep duration was self-reported (in 2004, 2006, and 2009) by answering the question, “How many hours each day do you usually sleep, including during both daytime and nighttime?” [21]. Sleep duration was divided into three categories: short sleep (≤6 h/day), normal sleep duration (>6 and ≤9 h/day), and long sleep (>9 h/day).

Height and body weight were measured by trained health workers based on a standard protocol recommended by the World Health Organization. Weight in lightweight in clothing was measured to the nearest 0.01 kg on a calibrated beam scale, and height was measured to the nearest 0.1 cm without shoes using a portable stadiometer. The details have been described in our previous study [20]. BMI was calculated by the formula weight (kg)/height^2^ (m), and divided into four categories based on the guideline for prevention and control of overweight and obesity in Chinese adults [22], which are underweight—BMI <18.5 kg/m^2^, normal weight—18.5 kg/m^2^ ≤ BMI < 24.0 kg/m^2^, overweight—24.0 kg/m^2^ ≤ BMI < 28.0 kg/m^2^, and obese—BMI ≥28.0 kg/m^2^ [22].

### 2.3. Dietary Consumption

At the individual level, dietary intake was assessed using 24-h recalls over three consecutive days. Individuals were asked to report all foods and beverages (measured in grams) consumed at home and away from home on a 24-h recall basis [23]. The dietary assessment method used in the study has been previously validated against double-labeled water for energy intake [24]. At the household level, dietary intake was weighed via a food inventory over the same three-day period. The amount of each dish prepared at home was estimated from the household inventory and the proportion of each dish consumed by each individual. Before the household interview, all the interviewers had three days of training on the collection of dietary data for the survey [2]. The detailed dietary data collection has been described elsewhere [20]. In the present study, we used the dietary data at the individual level to identify dietary patterns.

### 2.4. Covariates

Sociodemographic and lifestyle factors were collected and included as covariates in our analysis. The urbanization levels (low, medium, high) were based on the tertiles of a multidimensional twelve-component urbanization index [20]. The three education categories were low (≤primary school), medium (junior middle school), and high (≥high middle school). Smoking status was categorized into three groups: nonsmokers, ex-smokers, and current smokers. Physical activity level (metabolic equivalent of task (MET)/week) was estimated based on self-reported activities, including occupational, domestic, transportation, and leisure-time physical activities [25]. Blood pressure was measured three times after a 10 min seated test, using standard mercury sphygmomanometers with regular adult cuffs. Participants were identified as having diabetes and stroke if they self-reported to be physician-diagnosed of these diseases. Based on the question of “In last year, did you drink beer or any other alcoholic beverage?”, alcohol consumption was allocated to two categories (Yes/No).

### 2.5. Statistical Analysis

We identified dietary patterns using factor analysis. The detailed method of factor analysis has been described in our previous studies [10,20]. In brief, dietary patterns were identified based on eigenvalue (>1), scree plots, factor interpretability, and the variance explained (>5%). Factors were rotated with varimax to improve the interpretability of factors and minimize the correlation between factors. Participants were assigned a pattern-specific factor score, which was calculated as the sum of the products of the factor-loading coefficients and standardized daily intake of each food associated with that pattern. Factor loadings were included in the calculation of pattern scores.

Based on the dietary patterns constructed, we used group-based trajectory modeling to identify the trajectories of dietary pattern scores between 1991 and 2009. This latent class model approach identified subgroups within the study population that share a similar underlying trajectory in dietary patterns. The analysis was conducted using a user-written “traj” command in Stata, which is similar to the “proc traj” in SAS [16]. Survey year was used as a time variable to model the trajectory of dietary patterns. The following criteria were used to determine the number of trajectory groups: (1) the smallest Bayesian information criterion (BIC) and (2) each group should have at least 5% of the participants [17,18]. To estimate the association of trajectory groups to BMI, trajectory group membership was included as an independent variable in multiple linear regression models in examining predictors of sleep duration in 2009. The same approach was performed in predicting trajectory dietary patterns against BMI in 2009. There were three models used in the analysis: Model 1 was adjusted for age and gender, Model 2 was further adjusted for education, income, urbanization, smoking, alcohol drinking, and physical activity, and Model 3 was further adjusted for BMI and hypertension.

We also used a multivariable mixed linear model to assess the association between trajectories of dietary patterns with sleep duration or BMI. The models treated sociodemographic and lifestyle factors as time-varying covariates. All statistical analyses were conducted using STATA 16.1 (Stata Corporation, College Station, TX, USA).

## 3. Results

### 3.1. Dietary Patterns

We identified two dietary patterns, named traditional and modern dietary patterns. Table S1 shows the factor loading of the two dietary patterns. Rice, pork, fish, poultry, and fresh vegetable were positively and heavily loaded in the traditional dietary pattern, while wheat, whole grain, and deep-fried products were negatively loaded. Deep-fried products, milk, fruit, egg, fast food, and cake were positively loaded in the modern dietary pattern, while rice and salted vegetable were negatively loaded.

### 3.2. Trajectories of Dietary Patterns

The selection process for the trajectory groups of dietary patterns is shown in Table S2a,b. Of a total of 10,495 participants, we identified four trajectory groups of the traditional dietary pattern during the 18 years: Group 1 (*n* = 1030, 15.0%) had an initial low intake with a moderate increase but remained the lowest across all the survey years (named “low and rapid increase”); Group 2 (*n* = 1508, 22.3%) had a medium initial intake with a slight increase thereafter (named “medium and slow increase”); Group 3 (*n* = 1473, 22.3%) had the highest intake and remained high throughout the entire survey (named “high and stable”); Group 4 (*n* = 2932, 41.4%) had a high initial intake in 1991 and slightly decreased thereafter (named “high and slow decrease”).

**Figure 1:**
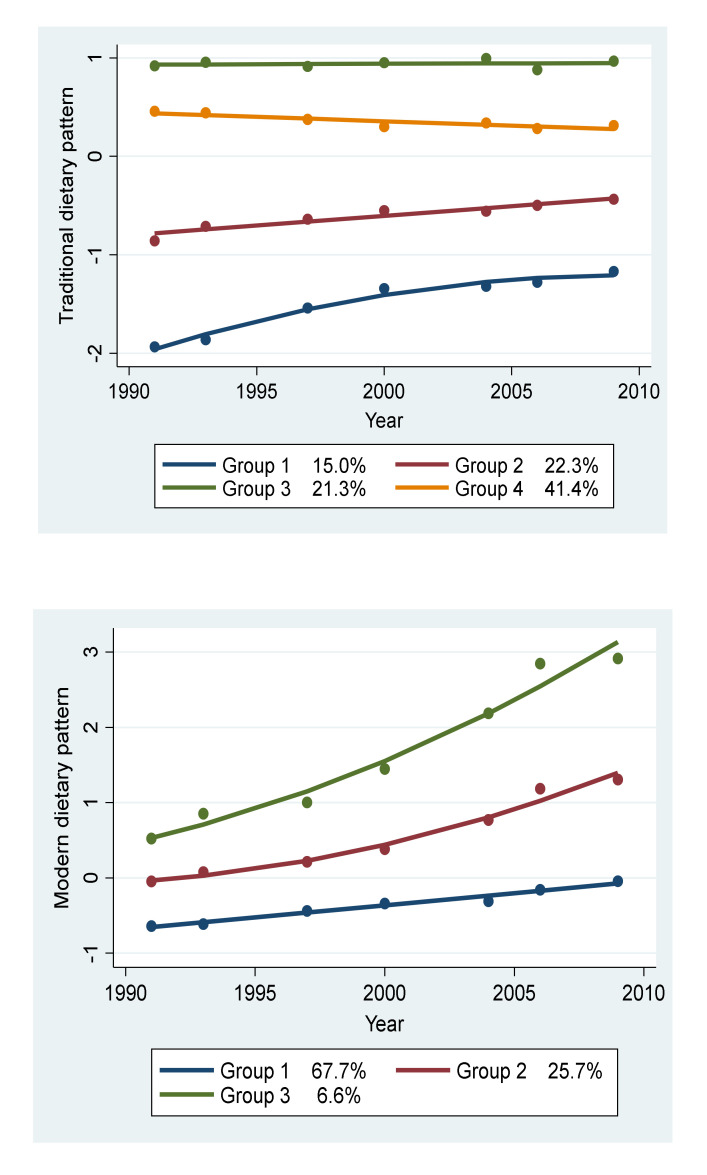
Dietary pattern trajectory groups between 1991 and 2009 among adults participating in the China Health and Nutrition Survey (*n* = 10,495)*. * All the included participants had at least three waves of dietary intake data.

Three trajectory groups of the modern dietary pattern were identified: Group 1 (*n* = 4864, 67.7%) had the lowest intake initially, with a slight increase thereafter, but it remained the lowest across all the survey years (named “low and slow increase”); Group 2 (*n* = 1689, 25.7%) had an initial medium intake and a moderate increase thereafter (named “medium and moderate increase”); Group 3 had the highest intake initially and rapidly increased throughout the entire survey (named “high and rapid increase”; Figure 1).

### 3.3. Participants’ Characteristics by Trajectory Groups

The participants in the four traditional dietary pattern trajectory groups had distinct characteristics profiles, as shown in Table 1. Significant differences in age, sex, education, and urbanization among four traditional dietary pattern trajectory groups were observed. Significant differences in health behavior factors (including smoking, alcohol drinking, tea consumption, physical activity, and sleep levels) and health conditions (including obesity, hypertension, diabetes, and stroke) were also observed among the four traditional dietary pattern trajectory groups. The proportion of men was the largest in Group 3 (64.9%). Participants in Group 3 had the youngest mean age (52.1). The majority of participants with a low education level were in Group 1 (56.8%), and 60.9% of participants who lived in high urbanization were in Group 3. Participants in Group 4 had the lowest physical activity level (mean MET, 118.8/week). Additionally, 80.9% of participants in Group 3 had sleep between 6 and 9 h.

The participants in the three modern dietary pattern trajectory groups also had distinct characteristics profiles, as shown in Table 2. Significant differences in sex, education, and urbanization among three modern dietary pattern trajectory groups were observed. Significant differences in health behavior factors (including smoking, alcohol drinking, tea consumption, physical activity, and sleep levels) and health conditions (including obesity and diabetes) were also observed among the three modern dietary pattern trajectory groups. The proportion of men was the largest in Group 3 (60.8%). The majority of participants with a low education level were in Group 1 (55.0%). Additionally, 55.2% of participants had a high education level, and 86.8% of participants living in high urbanization were in Group 3. Participants in Group 3 had the highest proportion of obesity (50.4%) and the highest proportion of sleep duration between 6 and 9 h (82.9%).

Marginal mean sleep duration by the trajectory groups of both dietary patterns over the years is shown in Figure S1. Sleep hours decreased across trajectory groups of both dietary patterns across the years. Group 1 in the traditional dietary pattern had the highest marginal mean sleep hours compared with the other three trajectory groups across the years. Group 1 in the modern dietary pattern had the highest marginal mean sleep hours, while Group 3 had the lowest marginal mean sleep hours across the years. The marginal mean of fat intake by the trajectories of both dietary patterns is shown in Figure S2. It can be seen that Group 1 in the traditional dietary pattern had the lowest marginal mean fat intake. Group 1 in the modern dietary pattern had the lowest fat intake throughout the entire survey, while Group 3 had the highest and most rapid increase in fat intake across the years.

### 3.4. Trajectory of Dietary Patterns and Sleep Duration

The associations between the trajectories of both dietary patterns and sleep duration in 2009 are shown in Table 3. There were significant differences in sleep duration among the three trajectories of the modern dietary pattern. Compared with Group 1, Group 2 (β = −0.30; 95% CI: −0.36, −0.23) and Group 3 (β = −0.39; 95% CI: −0.52, −0.27) were significantly associated with a lower sleep duration after adjusting for age and gender (Model 1). After adjusting for other potential covariates (Model 2), the modern dietary patterns in Group 2 (β = −0.20; 95% CI: −0.28, 0.13) and Group 3 (β = −0.26, 95% CI: −0.40, −0.13) were significantly associated with lower sleep duration. This association remained unchanged after further adjusting for BMI and hypertension. Adjusting for energy intake did not change the above association (data not shown). In addition, no significant interactions between residence, education, and trajectory of dietary patterns were found (Figure S3). There were no associations between trajectory groups of the traditional dietary pattern and sleep duration.

### 3.5. Trajectory of Dietary Patterns and BMI

**Figure 2:**
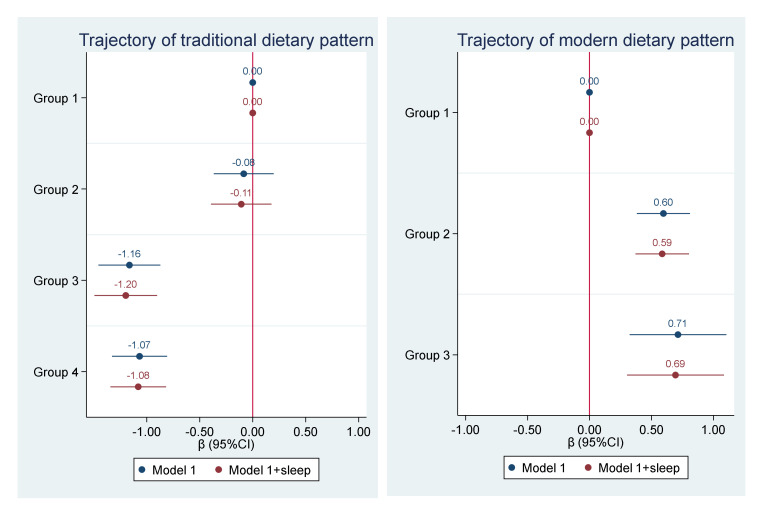
Association between trajectories of dietary patterns (between 1991 and 2009) and BMI in 2009*. * Model 1 is adjusted for age, sex, education, income, urbanization, smoking, alcohol drinking, and physical activity and hypertension. Sleep was treated as a continuous variable in the model. Trajectories of the traditional dietary pattern: Group 1 (low and rapid increase), Group 2 (medium and slow increase), Group 3 (high and stable), Group 4 (high and slow decrease). Trajectories of the modern dietary pattern: Group 1 (low and slow increase), Group 2 (medium and moderate increase), Group 3 (high and rapid increase).

The multivariable-adjusted association between the trajectories of both dietary patterns and BMI in 2009 is shown in Figure 2. Compared with Group 1 in the traditional pattern, Group 3 (β = −1.16; 95% CI: −1.46, −0.87) and Group 4 (β = −1.07; 95% CI: −1.33, −0.81) had significant lower BMI. Compared with the modern pattern in Group 1, Group 2 (β = 0.60; 95% CI: 0.38, 0.82) and Group 3 (β = 0.71; 95% CI: 0.32, 1,10) had significant higher BMI. However, there was no mediation effect of sleep on the relationship between the trajectories of dietary patterns and BMI, as adjusting for sleep in the multivariable model did not change the regression coefficients. The findings were also observed among nonsmokers (Figure S4).

## 4. Discussion

To our best knowledge, this is the first study to identify trajectories of dietary patterns and evaluate their associations with sleep duration and BMI in a large cohort of the Chinese population that has been followed for 18 years. Four trajectories of the traditional dietary pattern and three trajectories of the modern dietary pattern were identified. Participants who followed the high and rapid increase trajectory of the modern dietary pattern (Group 3) had the shortest sleep duration. Participants with a high intake of the traditional dietary pattern throughout the entire survey between 1991 and 2009 (Group 3) had the lowest BMI, while participants with the high and rapid increase trajectory of the modern dietary pattern (Group 3) had the highest BMI. Trajectories of the traditional dietary pattern were not associated with sleep duration. Sleep duration was not found to be a mediator in the relationship between the trajectories of dietary patterns and BMI.

### 4.1. Demographic Characteristics among the Trajectories of Dietary Patterns

We found significant differences in demographic characteristics between traditional and modern dietary patterns. Relatively young men who live in highly urbanized areas tend to maintain a traditional dietary pattern (i.e., Group 3 of the traditional dietary trajectory, high and stable). However, they were more likely to be current smokers and frequent alcohol consumers. This is surprising but understandable as in the Chinese culture, smoking and alcohol consumption are perceived as traits of a rich person and symbolic of a high class, particularly in men. Likewise, men with high education, living at an urbanization level, who were current smokers and alcohol consumers, were more likely to adopt a modern dietary pattern (i.e., Group 3 of the modern dietary pattern, high and rapid increase). They were more likely to be overweight or obese and had the lowest level of physical activity. This may indicate a synergistic effect of an unhealthy diet and a low level of physical activity. This finding is in line with previous studies that reported a synergistic effect of poor diet and sedentary behavior in escalating cardiometabolic risk [26].

### 4.2. Trajectories of Dietary Patterns and Sleep Duration

A significant positive association between the high and rapid increase trajectory group of the modern dietary pattern and short sleep was highlighted in our study. This result is consistent with previous findings on the association between unhealthy food intake and short sleep duration [6,7]. More importantly, we have further investigated the association between the trajectories of dietary patterns, short sleep, and BMI to determine if short sleep was on the pathway between the modern dietary pattern and BMI. However, no significant mediation effect was found, as suggested by the very small change of the regression coefficients in models with and without the adjustment of sleep duration. This is an interesting finding, as the sleep–obesity mechanism mediated by dietary behavior has often been discussed in previous studies. It has been reported that dietary patterns partially explained the association between short sleep and obesity [27]. However, whether sleep can partly explain the association between diet and obesity has not been well studied. One possible reason might be that the impact of diet on BMI is dominant over sleep duration. Another possibility of the null mediation effect of sleep on dietary patterns and BMI may be due to the fact that the prevalence of short sleep duration is lower in China as compared with other Western countries [28].

### 4.3. Trajectories of Dietary Patterns and BMI

The existing literature has shown that a high intake of the traditional dietary pattern is inversely associated with BMI in the Chinese population, whereas a modern dietary pattern shows a positive association [14,29]. The possible mechanism can be attributed to the protective effect of lower energy foods and higher fiber foods (highly loaded in the traditional dietary pattern) on obesity [30]. Similarly, higher fat and energy foods (highly loaded in the modern dietary pattern) have been demonstrated to increase the risk of obesity [31]. Our results are in line with these results and provide further novel evidence regarding how the dietary patterns change in the population over time and how they affect BMI. Specifically, our results highlighted that a high intake of the traditional dietary pattern throughout the entire survey was associated with the lowest BMI, and the difference was substantial (β = −1.07; 95% CI: −1.33, −0.81), while a high and rapid increase trajectory of the modern dietary pattern was associated with the highest BMI (β = 0.71; 95% CI: 0.32, 1,10). Our results suggest the importance of maintaining a healthy dietary pattern in keeping a normal BMI. Similar trajectory analysis of food intake (e.g., the Mediterranean diet score) and its associations with diabetes [32] and hypertension [18] have been examined in a limited number of studies in China. Due to the different methods of constructing dietary patterns, the trajectory of the dietary patterns cannot be compared directly. However, the relatively stable intake of the traditional dietary pattern and the increased intake of the modern dietary pattern in our study were similar to a previous study using CHNS data [14]. The previous study examined the mean intake of the traditional and modern dietary patterns between 1991 and 2009 and found the former was stable, but the latter was increased [14]. However, what we found here is that the different levels of the intake of the traditional pattern were relatively stable.

The strength of the present study is that comprehensive analyses were conducted using longitudinal data from a large population sample by using a multistage and random cluster process. Dietary data collection, including individual and consecutive 3-day recall methods, significantly improved the accuracy of the recalled information and hence our results. In addition, by using the dietary pattern trajectory approach, our results provide significant insights into the relationship between different dietary pattern trajectories, sleep duration, and BMI. However, there are some limitations that need to be recognized. Firstly, data on dietary intake and sleep duration were self-reported, which may induce measurement bias. However, self-reported sleep measures are the most commonly used measure in population surveys and can be complementary to objective measures of sleep [33]. In our study, sleep duration may be overestimated. However, short sleep duration has been demonstrated with an increased risk of hypertension among participants attending CHNS [21]. Secondly, we did not have information on sleep quality, the use of sleep medicine, or supplement intake. Thus, we were unable to examine whether the association between the trajectories of dietary patterns and obesity was mediated by sleep quality. Thirdly, we used self-reported physical activity. As physical activity is associated with both sleep quality [34] and dietary intake, the validity of the measure of physical activity is vital in the study of the association between diet and sleep. However, self-reported physical activity has been widely used in large population-based studies. In the study, we have information on four types of physical activities, including domestic, occupational, transportation, and leisure-time physical activities.

## 5. Conclusions

In conclusion, we found that a high and rapid increase of the modern dietary pattern was associated with shorter sleep duration but higher BMI. The association between the modern dietary pattern and BMI is unlikely to be mediated by sleep duration. Given the rapid increase of the modern dietary pattern in the Chinese population in the previous decades, promoting and maintaining healthy eating is important to reduce the risk of obesity and short sleep, as well as other related diseases.

## Figures and Tables

**Table 1:** Sample characteristics by trajectories of the traditional dietary pattern in 2009 (*n* = 6943)*.

	Group 1: Low and Rapid Increase	Group 2: Medium and Slow Increase	Group 3: High and Stable	Group 4: High and Slow Decrease	*p*-Value
	***n* = 1030**	***n* = 1508**	***n* = 1473**	***n* = 2932**	
Age (years), mean (SD)	53.7 (12.9)	54.9 (13.3)	52.1 (11.9)	55.2 (13.7)	<0.001
Sex, *n* (%)					<0.001
Men	516 (50.1%)	650 (43.1%)	956 (64.9%)	1242 (42.4%)	
Women	514 (49.9%)	858 (56.9%)	517 (35.1%)	1690 (57.6%)	
Education, *n* (%)					<0.001
Low	585 (56.8%)	704 (46.7%)	541 (36.8%)	1429 (48.9%)	
Medium	317 (30.8%)	478 (31.7%)	555 (37.8%)	876 (30.0%)	
High	128 (12.4%)	325 (21.6%)	374 (25.4%)	618 (21.1%)	
Urbanization, *n* (%)					<0.001
Low	421 (40.9%)	286 (19.0%)	151 (10.3%)	196 (6.7%)	
Medium	361 (35.0%)	580 (38.5%)	425 (28.9%)	1317 (44.9%)	
High	248 (24.1%)	642 (42.6%)	897 (60.9%)	1419 (48.4%)	
Smoking, *n* (%)					<0.001
Nonsmoker	679 (65.9%)	1034 (68.6%)	849 (57.7%)	2107 (71.9%)	
Ex-smokers	53 (5.1%)	50 (3.3%)	60 (4.1%)	97 (3.3%)	
Current smokers	298 (28.9%)	423 (28.1%)	563 (38.2%)	727 (24.8%)	
Alcohol drinking, *n* (%)	330 (32.3%)	458 (30.4%)	670 (45.8%)	852 (29.2%)	<0.001
Tea consumption (cups/d), *n* (%)					<0.001
None	784 (76.2%)	1066 (70.8%)	795 (54.5%)	1874 (64.1%)	
<2 cup/d	67 (6.5%)	132 (8.8%)	177 (12.1%)	312 (10.7%)	
2–3.9 cup/d	91 (8.8%)	154 (10.2%)	204 (14.0%)	360 (12.3%)	
≥4 cup/d	87 (8.5%)	153 (10.2%)	283 (19.4%)	378 (12.9%)	
Physical activity (MET, hour/week), mean (SD)	138.2 (116.8)	138.0 (121.5)	127.0 (102.2)	118.8 (104.6)	<0.001
BMI (kg/m^2^), mean (SD)	24.0 (3.4)	24.2 (3.5)	23.2 (3.2)	23.1 (3.5)	<0.001
BMI >24 (kg/m^2^), *n* (%)	468 (48.0%)	712 (48.9%)	501 (36.4%)	1022 (36.5%)	<0.001
Hypertension, *n* (%)	295 (29.7%)	514 (34.8%)	382 (27.6%)	828 (29.2%)	<0.001
Diabetes, *n* (%)	22 (2.1%)	61 (4.1%)	34 (2.3%)	91 (3.1%)	0.012
Stroke, *n* (%)	25 (2.4%)	29 (1.9%)	13 (0.9%)	39 (1.3%)	0.008
Sleep duration (hour/d), mean (SD)	8.1 (1.2)	7.9 (1.2)	7.9 (1.1)	7.9 (1.2)	<0.001
Sleep duration, *n* (%)					<0.001
7–9 h	794 (77.1%)	1174 (77.9%)	1191 (80.9%)	2252 (76.8%)	
≤6 h	86 (8.3%)	163 (10.8%)	149 (10.1%)	333 (11.4%)	
>9 h	150 (14.6%)	171 (11.3%)	133 (9.0%)	347 (11.8%)	

* Data are presented as mean (SD) for continuous measures, and n (%) for categorical measures.

**Table 2:** Sample characteristics by trajectory of modern dietary pattern in 2009 (*n* = 6943) *.

	Group 1: Low and Slow Increase	Group 2: Medium and Moderate Increase	Group 3: High and Rapid Increase	*p*-Value
	***n* = 4864**	***n* = 1689**	***n* = 390**	
Age (years), mean (SD)	54.4 (13.3)	53.9 (12.8)	54.0 (13.6)	0.41
Sex, *n* (%)				<0.001
Men	2274 (46.8%)	856 (50.4%)	234 (60.8%)	
Women	2584 (53.2%)	844 (49.6%)	151 (39.2%)	
Education, *n* (%)				<0.001
Low	2666 (55.0%)	531 (31.3%)	62 (16.1%)	
Medium	1552 (32.0%)	564 (33.2%)	110 (28.6%)	
High	629 (13.0%)	604 (35.6%)	212 (55.2%)	
Urbanization, *n* (%)				<0.001
Low	959 (19.7%)	90 (5.3%)	5 (1.3%)	
Medium	2245 (46.2%)	392 (23.1%)	46 (11.9%)	
High	1654 (34.0%)	1218 (71.6%)	334 (86.8%)	
Smoking, *n* (%)				<0.001
Nonsmoker	3287 (67.7%)	1148 (67.6%)	234 (60.8%)	
Ex-smokers	164 (3.4%)	67 (3.9%)	29 (7.5%)	
Current smokers	1405 (28.9%)	484 (28.5%)	122 (31.7%)	
Alcohol drinking, *n* (%)	1462 (30.3%)	652 (38.5%)	196 (51.0%)	<0.001
Tea consumption (cups/d)				<0.001
None	3357 (69.4%)	1003 (59.3%)	159 (41.3%)	
<2 cup/d	449 (9.3%)	186 (11.0%)	53 (13.8%)	
2–3.9 cup/d	515 (10.6%)	229 (13.5%)	65 (16.9%)	
≥4 cup/d	519 (10.7%)	274 (16.2%)	108 (28.1%)	
Physical activity (MET, hour/week), mean (SD)	135.0 (116.0)	110.8 (95.3)	106.3 (77.8)	<0.001
BMI (kg/m^2^), mean (SD)	23.2 (3.4)	24.1 (3.5)	24.2 (3.6)	<0.001
BMI >24 (kg/m^2^), *n* (%)	1733 (37.6%)	785 (48.2%)	185 (50.4%)	<0.001
Hypertension, *n* (%)	1369 (29.3%)	530 (32.2%)	120 (31.7%)	0.073
Diabetes, *n* (%)	104 (2.1%)	80 (4.7%)	24 (6.2%)	<0.001
Stroke, *n* (%)	74 (1.5%)	24 (1.4%)	8 (2.1%)	0.63
Sleep (hour/week), mean (SD)	8.0 (1.2)	7.7 (1.1)	7.6 (1.1)	<0.001
Sleep duration, *n* (%)				<0.001
7–9 h	3731 (76.8%)	1361 (80.1%)	319 (82.9%)	
≤6 h	469 (9.7%)	217 (12.8%)	45 (11.7%)	
>9 h	658 (13.5%)	122 (7.2%)	21 (5.5%)	

* Data are presented as mean (SD) for continuous measures and n (%) for categorical measures.

**Table 3:** Association between trajectories (between 1991 and 2009) of dietary patterns and sleep duration among adults participating in the China Health and Nutrition Survey in 2009*.

**Traditional Pattern**				
	Group 1: Low and rapid increase	Group 2: Medium and slow increase	Group 3: High and stable	Group 4: High and slow decrease
Model 1	Reference	−0.18 (−0.27, −0.08)	−0.21 (−0.31, −0.11)	−0.15 (−0.24, −0.06)
Model 2	Reference	−0.02 (−0.12, 0.08)	0.00 (−0.10, 0.11)	0.02 (−0.07, 0.12)
Model 3	Reference	−0.01 (−0.11, 0.09)	0.01 (−0.09, 0.12)	0.02 (−0.07, 0.12)
Model 4	Reference	0.01 (−0.12, 0.13)	0.07 (−0.06, 0.20)	0.02 (−0.09, 0.13)
**Modern Pattern**				
	Group 1: Low and slow increase	Group 2: Medium and moderate increase	Group 3: High and rapid increase	
Model 1	Reference	−0.30 (−0.36, −0.23)	−0.39 (−0.52, −0.27)	
Model 2	Reference	−0.20 (−0.28, −0.13)	−0.26 (−0.40, −0.13)	
Model 3	Reference	−0.20 (−0.28, −0.13)	−0.26 (−0.40, −0.12)	
Model 4	Reference	−0.18 (−0.27, −0.09)	−0.15 (−0.33, 0.02)	

* Values are regression coefficients and 95% confidence intervals. Model 1 is adjusted for age and gender. Model 2 is further adjusted for education, income, urbanization, smoking, alcohol drinking, and physical activity. Model 3 is further adjusted for BMI (continuous) and hypertension. Model 4 is Model 3 among nonsmokers.

## Supplementary Materials

The following are available online at https://www.mdpi.com/2072-6643/12/8/2245/s1. Table S1: Factor loadings of the dietary patterns. Table S2a: Selection process for the trajectory groups of the dietary patterns. Table S2b: Selection process for the trajectory groups of the dietary patterns. Figure S1: Marginal mean sleep duration by the trajectory groups of the dietary patterns. Figure S2: Marginal mean fat intake by the trajectory groups of the modern dietary pattern. Figure S3: Association between the trajectories of dietary patterns (between 1991 and 2009) and sleep duration in 2009 by urbanization and education levels. Figure S4: Association between the trajectories of the dietary patterns (between 1991 and 2009) and BMI in 2009 among nonsmokers.
