# Supplementary material for: Trajectories of Dietary Patterns, Sleep Duration, and Body Mass Index in China: A Population-Based Longitudinal Study from China Nutrition and Health Survey, 1991–2009
Source: Nutrients. 2020 Jul 27;12(8):2245. doi: 10.3390/nu12082245 (PMC7469025; doi:10.3390/nu12082245)
Supplement: Supplementary file 1 [file nutrients-12-02245-s001.pdf]

Table S1: Factor loadings of dietary patterns*

Variable	Traditional	Modern
Wheat	**-0.728**	0.068
Rice	**0.711**	**-0.387**
Pork	**0.475**	**0.217**
Whole grain	**-0.462**	-0.042
Fish	**0.418**	0.183
Poultry	**0.301**	**0.205**
Dry tofu	**0.292**	0.031
Fresh vegetable	**0.243**	-0.195
Beef	**0.237**	0.161
Offal	**0.229**	0.035
Tubers	-0.183	-0.113
Spirit	0.123	0.110
Bean thread noodle	-0.092	0.064
Tofu	-0.057	0.055
Wine	0.018	0.018
Fruit	0.080	**0.442**
Soymilk	-0.077	**0.420**
Eggs	-0.031	**0.418**
Deep-fried products	**-0.204**	**0.408**
Milk	0.047	**0.399**
Fast food	-0.023	**0.384**
Cake	-0.015	**0.304**
Fungus	0.152	**0.283**
Beer	0.087	**0.236**
Nuts	0.096	**0.229**
Shrimp	0.120	**0.228**
Salted vegetable	0.078	**-0.208**
Lamb	0.003	0.199
Yoghurt	0.037	0.151
Others	0.027	0.116
Beverage	0.038	0.109
Sugar	0.026	0.103
Legume	-0.017	-0.102
Milk powder	0.016	0.056
Fresh bean	0.008	0.032

**bold: Factor loadings of >|0.20| represent the foods which most strongly related to the identified factor.*

Table S2a: Selection process for the trajectory groups of traditional dietary patterns

Number of groups	Trajectory shapes (orders)	BIC	Proportion of groups (%)			
			Group 1	Group 2	Group 3	Group 4
1	1	-73111.49	100			
2	1 1	-57992.11	33.3	66.7		
3	1 1 1	-55529.93	18.7	26.9	54.4	
4	1 1 1 1	-54493.09	14.8	21.5	41.3	22.4
**4 ***	**2 1 1 1**	**-54450.03**	**15.0**	**22.3**	**21.3**	**41.4**
4	3 1 1 1	-54455.52	15.0	22.3	21.3	41.4
3	1 1 2	-55528.24	18.7	26.8	54.5	
3	1 2 1	-55535.38	18.7	26.9	54.4	
3	1 2 2	-55533.29	18.7	26.8	54.5	
3	2 1 1	-55482.41	19.0	26.8	54.2	
3	2 1 2	-55480.79	18.9	26.8	54.3	
3	2 2 1	-55487.73	18.9	26.8	54.3	
3	2 2 2	-55485.91	18.9	54.4	26.7	

Trajectory shapes: 1 = linear; 2 = quadratic; c = cubic
BIC: Bayesian information criterion
*Final trajectory used in the study

Table S2b: Selection process for the trajectory groups of modern dietary patterns

Number of groups	Trajectory shapes (orders)	BIC	Proportion of groups (%)			
			Group 1	Group 2	Group 3	Group 4
1	1	-70456.33	100			
2	1 1	-63834.43	79.1	20.9		
3	1 1 1	-62516.73	68.2	25.3	6.5	
4	1 1 1 1	-62099.65	25.6	2.1	64.8	7.6
3	1 1 2	-62483.09	68.1	25.2	6.7	
3	1 1 3	-62488.70	68.1	25.2	6.7	
3	1 2 1	-62435.30	67.8	25.6	6.5	
3	1 2 2	-62433.11	67.8	25.6	6.6	
**3 ***	**1 2 3**	**-62430.78**	**67.7**	**25.7**	**6.6**	
3	2 1 1	-62435.30	25.6	67.8	6.5	
3	2 1 2	-62433.11	25.6	67.8	6.6	
3	2 2 1	-62438.69	25.6	67.9	6.5	
3	2 2 2	-62435.71	67.8	25.6	6.6	

Trajectory shapes: 1 = linear; 2 = quadratic; 3= cubic

BIC: Bayesian information criterion

*Final trajectory used in the study

Figure S1: Marginal mean sleep duration by trajectory groups of dietary patterns*

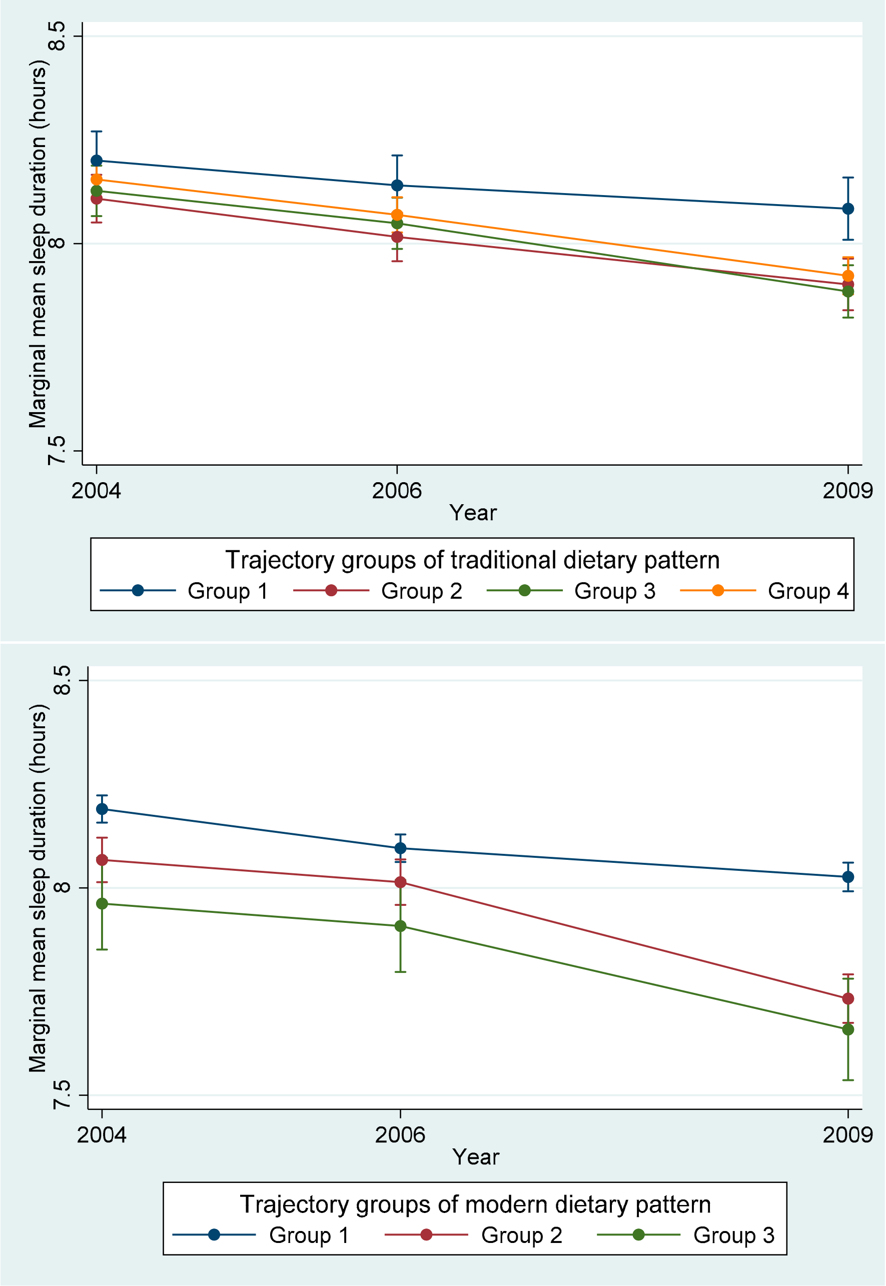

*Values are adjusted for age and gender using mixed command in Stata. *marginsplot* command was used to generate the figure.

Trajectory of traditional dietary patterns: Group 1 (Low and rapid increase), Group 2 (Medium and slow increase), Group 3 (High and stable), Group 4 (High and slow decrease).
Trajectory of modern dietary patterns: Group 1 (Low and slow increase), Group 2(Medium and moderate increase), Group 3 (High and rapid increase).

Figure S2: Marginal mean fat intake by trajectory groups of modern dietary pattern*

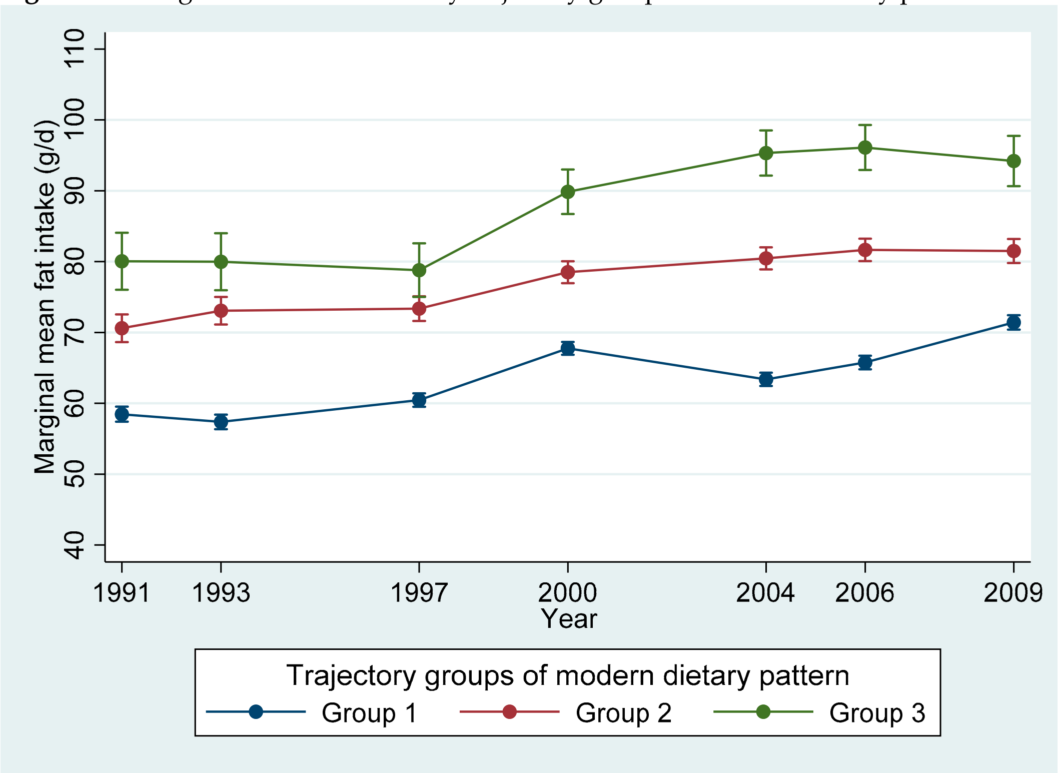

**Values are adjusted for age and gender using mixed command in Stata. marginsplot command was used to generate the figure.*
Trajectory of modern dietary patterns: Group 1 (Low and slow increase), Group 2(Medium and moderate increase), Group 3 (High and rapid increase).

Figure S3 Association between trajectory of dietary patterns (between 1991 and 2009) and sleep duration in 2009 by urbanization and education levels

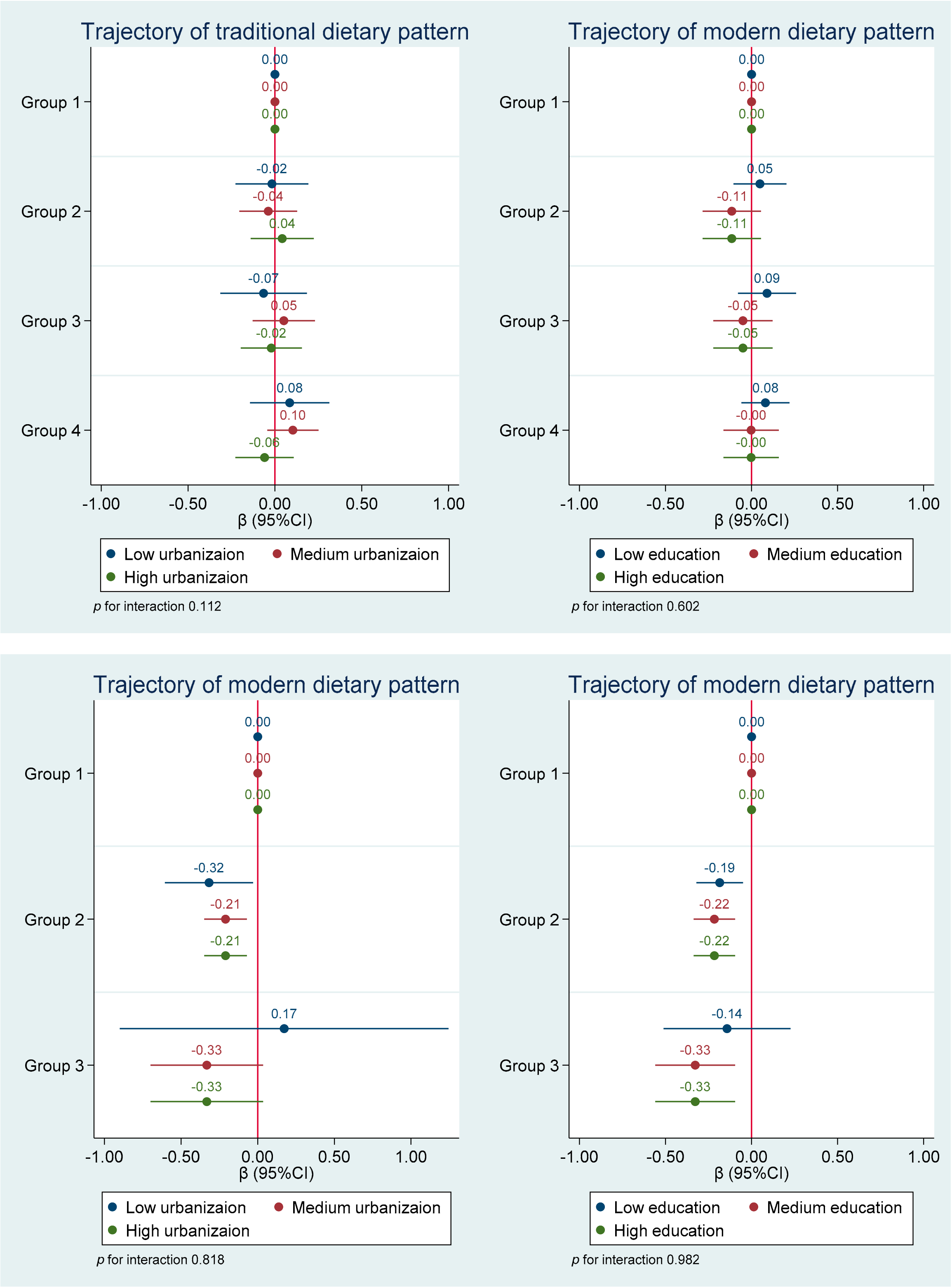

**Model adjusted for age, sex, education, income, urbanization, smoking, alcohol drinking and physical activity and hypertension. Stratification variables were not adjusted in the corresponding models.*

Trajectory of modern dietary patterns: Group 1 (Low and slow increase), Group 2(Medium and moderate increase), Group 3 (High and rapid increase).
Trajectory of modern dietary patterns: Group 1 (Low and slow increase), Group 2(Medium and moderate increase), Group 3 (High and rapid increase).

Figure S4. Association between trajectory of dietary patterns (between 1991 and 2009) and BMI in 2009 among non-smokers*

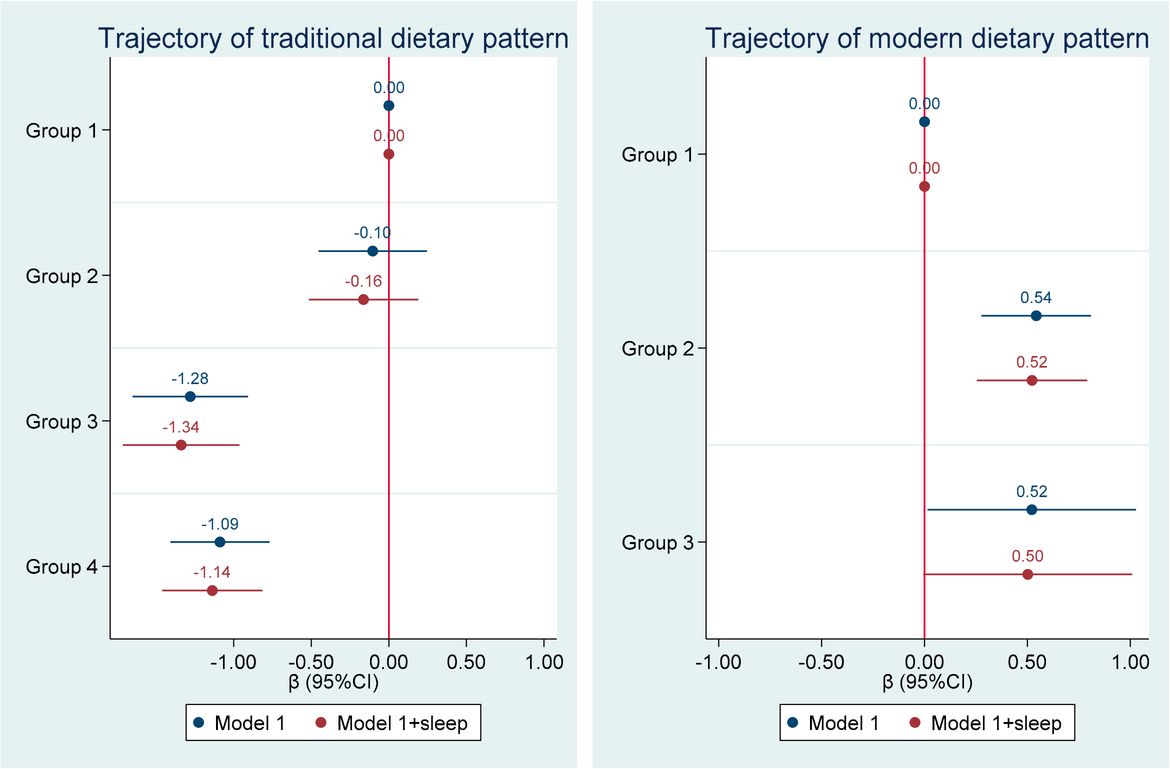

**Model 1 adjusted for age, sex, education, income, urbanization, smoking, alcohol drinking and physical activity and hypertension.*
Trajectory of traditional dietary patterns: Group 1 (Low and rapid increase), Group 2 (Medium and slow increase), Group 3 (High and stable), Group 4 (High and slow decrease).
Trajectory of modern dietary patterns: Group 1 (Low and slow increase), Group 2(Medium and moderate increase), Group 3 (High and rapid increase).
